# Detection of cancer stem cells by EMT-specific biomarker-based peptide ligands

**DOI:** 10.1038/s41598-021-01138-0

**Published:** 2021-11-17

**Authors:** Yi-An Chen, Cheau-Ling Ho, Min-Tzu Ku, Luen Hwu, Cheng-Hsiu Lu, Sain-Jhih Chiu, Wen-Yi Chang, Ren-Shyan Liu

**Affiliations:** 1 Department of Biomedical Imaging and Radiological Sciences, National Yang Ming Chiao Tung University, Taipei, 112 Taiwan; 2 Molecular and Genetic Imaging Core/Taiwan Mouse Clinic, National Comprehensive Mouse Phenotyping and Drug Testing Center, Taipei, 112 Taiwan; 3 PET center, Department of Nuclear Medicine, Taipei Veterans General Hospital, Taipei, 112 Taiwan; 4 Core Laboratory for Phenomics and Diagnostics, Kaohsiung Chang Gung Memorial Hospital, Kaohsiung, 833 Taiwan; 5 Department of Medical Research, Kaohsiung Chang Gung Memorial Hospital, Kaohsiung, 833 Taiwan; 6 Department of Nuclear Medicine, Cheng Hsin General Hospital, Taipei, 112 Taiwan

**Keywords:** Cancer, Biomarkers

## Abstract

The occurrence of epithelial-mesenchymal transition (EMT) within tumors, which enables invasion and metastasis, is linked to cancer stem cells (CSCs) with drug and radiation resistance. We used two specific peptides, F7 and SP peptides, to detect EMT derived cells or CSCs. Human tongue squamous carcinoma cell line-SAS transfected with reporter genes was generated and followed by spheroid culture. A small molecule inhibitor-Unc0642 and low-dose ionizing radiation (IR) were used for induction of EMT. Confocal microscopic imaging and fluorescence-activated cell sorting analysis were performed to evaluate the binding ability and specificity of peptides. A SAS xenograft mouse model with EMT induction was established for assessing the binding affinity of peptides. The results showed that F7 and SP peptides not only specifically penetrated into cytoplasm of SAS cells but also bound to EMT derived cells and CSCs with high nucleolin and vimentin expression. In addition, the expression of CSC marker and the binding of peptides were increased in tumors isolated from Unc0642/IR-treated groups. Our study demonstrates the potential of these peptides for detecting EMT derived cells or CSCs and might provide an alternative isolation method for these subpopulations within the tumor in the future.

## Introduction

Cancer stem cells (CSCs), the subpopulation of tumor cells, is commonly associated with aggressive tumor growth and increased risk of recurrence. CSCs exhibit stem-like features, like self-renewal and differentiation, which may cause higher resistance against chemo- and radiation therapy^1^. Induction of an epithelial-mesenchymal transition (EMT) in cancer cells leads to the acquisition of mesenchymal features, immune escape and maintenance of stemness^2^. Recent studies have demonstrated that the EMT programme is a critical regulator of the CSC phenotype, thereby permitting CSC-mediated clinical relapse^3^. It is essential to eradicate either tumor cell populations induced into EMT or CSCs-enriched populations for the improvement of clinical outcomes.

Squamous cell carcinoma is the most common oral cancer which presents a rapid clinical progression and a poor prognosis, attributing to the local recurrence, cervical lymph node metastasis and occasionally distant metastasis^4^. The main treatment for early-stage oral cancer is surgery, whereas chemotherapy plus radio-therapy are employed for treating patients with advanced cancer. However, the high heterogeneity of malignant tumors and the existence of cancer stem cells (CSCs) make it difficult to be killed through traditional treatment approaches^5^. Thus, developing effective methods for early detection of CSCs or EMT derived cells to provide more effective therapeutic strategies is our priority.

There are several sets of specific markers suitable to identify cancer cell populations enriched for CSCs and EMT derived cells. Nucleolin is a multifunctional phosphoprotein localized primarily in the nucleolus regulating rRNA transcription and ribosome biogenesis. Notably, cell surface nucleolin acts as a low-affinity receptor involved in cell differentiation, adhesion, angiogenesis and tumor development^6^, and the level of nucleolin are known to be increased in several types of cancers^7^. Increasing evidence has demonstrated that cell surface nucleolin can be a novel target for anticancer therapy^8,9^. Screening of phage display libraries has validated a peptide sequence DMPGTVLP, referred as F7 peptide, selectively binding to the surface nucleolin on MCF-7^10^. Vimentin, an intermediate filament protein, is responsible for maintaining cellular shape and integrity as well as stabilizing cytoskeletal interactions^11^. Recently, vimentin is also regarded as a standard biomarker for EMT. The level of vimentin in tumor cells correlates well with increased tumor growth, invasion and poor prognosis^3^. Likewise, a SP peptide (SAHGTSTGVPWP) that specifically binds cell surface vimentin is identified by screening a phage display peptide library on human vascular endothelial cells under hypoxic conditions^12,13^. Herein, F7 and SP peptides are potential biomarker-based peptides to detect CSCs or EMT derived cells.

In the current study, we generated two human tongue squamous carcinoma derived cell lines with the fluorescent and luciferase genes, SAS-EGFP-Fluc and SAS-E2-crimson-P2A::ttksr39 cells, to investigate the binding ability and specificity of F7 and SP peptides. A small molecule inhibitor-Unc0642 and low-dose ionizing radiation (IR) were used to simulate the cancer recurrence after chemotherapy and radiotherapy, thereby inducing EMT. The in vitro data showed that the F7 and SP peptides were capable of detecting EMT derived cells with high vimentin and nucleolin expressions. In addition, a tumor xenograft mouse model with EMT induction was established to evaluate the binding ability of F7 peptide and SP peptide. Our results suggest that F7 peptide and SP peptide can serve as a tool to detect CSCs or EMT derived cells.

## Results

### Evaluation of peptide binding activity to CSCs

**Figure 1:**
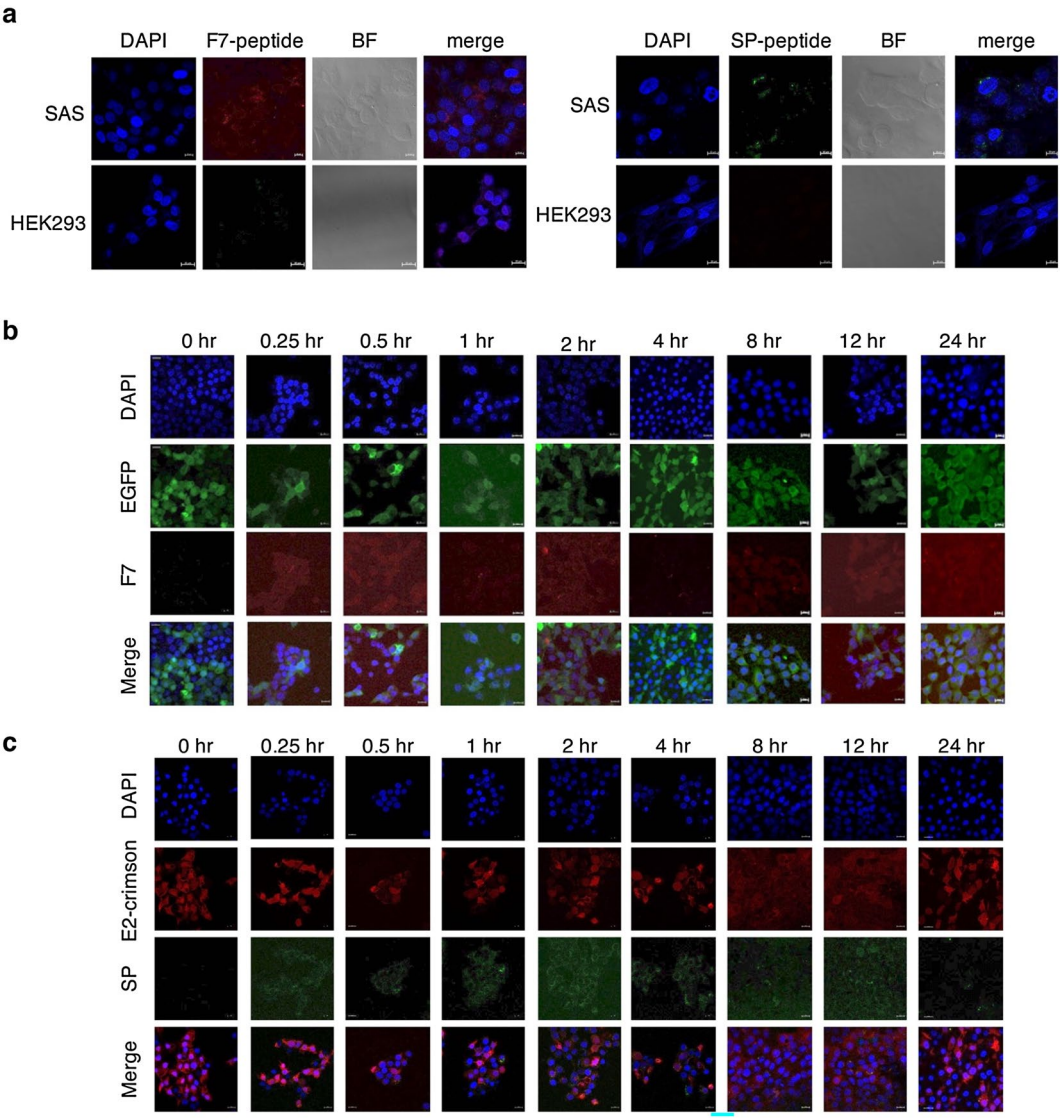
Evaluation of peptide binding activity to SAS cells. (**a**) IF analysis of F7 peptide and SP peptide binding to SAS cells. HEK293 cell that does not express vimentin and nucleolin served as a negative control. Red: Lissamine Rhodamine labeled F7 peptide, Green: FITC labeled SP peptide, Blue: DAPI; BF, bright field; Scale bar, 10 μm. (**b**) The kinetics of F7 peptide binding to SAS-EGFP-Fluc cells. Cells were incubated in the presence of 50 ng/μl peptides at 37 °C for the indicated periods of time. (**c**) The kinetics of SP peptide binding to SAS-E2-crimson-P2A::ttksr39 cells at indicated time points.

To track the EMT-derived cells both in vitro and in vivo, we transduced the reporter genes to the human tongue squamous carcinoma derived cell line-SAS using lentivirus, which were referred as SAS-EGFP-Fluc and SAS-E2-crimson-P2A::ttksr39 cells, respectively. The expression of E2-Crimson (red fluorescence) and EGFP fluorescence in stable cell lines were visualized under a confocal fluorescence microscopy (Fig. S1a). Fluorescence-activated cell sorting (FACS) analysis also revealed over 90% of stable lines exhibiting the higher fluorescence intensity compared to SAS-wt cells (Fig. S1b). SAS-EGFP-Fluc cells showed higher luciferase activity as demonstrated by luciferase assay (Fig. S1c). Furthermore, there was no significant difference in doubling time between SAS-wt, SAS-EGFP-Fluc and SAS-E2-crimson-P2A::ttksr39 cells, indicating that these transgenes did not affect cell growth (Fig. S1d). To evaluate whether F7 and SP peptides are able to bind to SAS cells, Lissamine Rhodamine (red fluorescence) labeled F7 peptide and FITC-labeled SP peptide, were used. As shown in Fig. 1a, the fluorescent signal derived from F7 and SP peptides were found in SAS cells that are known to express vimentin and nucleolin. HEK293 cells without detectable vimentin and nucleolin were used to be a negative control for the specificity of peptides. For further confirmation, the kinetics of F7 and SP peptide binding to SAS-EGFP-Fluc and SAS-E2-crimson-P2A::ttksr39 cells respectively were monitored at 37 °C for various periods of time. Within 30 min, either F7 peptide or SP peptide was observed to adhere to the cell membrane. One hour later, the fluorescent signal appeared in cytoplasm gradually and accumulated with the incubation time (Fig. 1b and c). At 24 h, F7 and SP peptides remained at the edge of nucleus and no longer moved. These results indicated that both F7 and SP peptides penetrated into cytoplasm of SAS cells specifically and then accumulated within cells over time rather than bound randomly.

**Figure 2:**
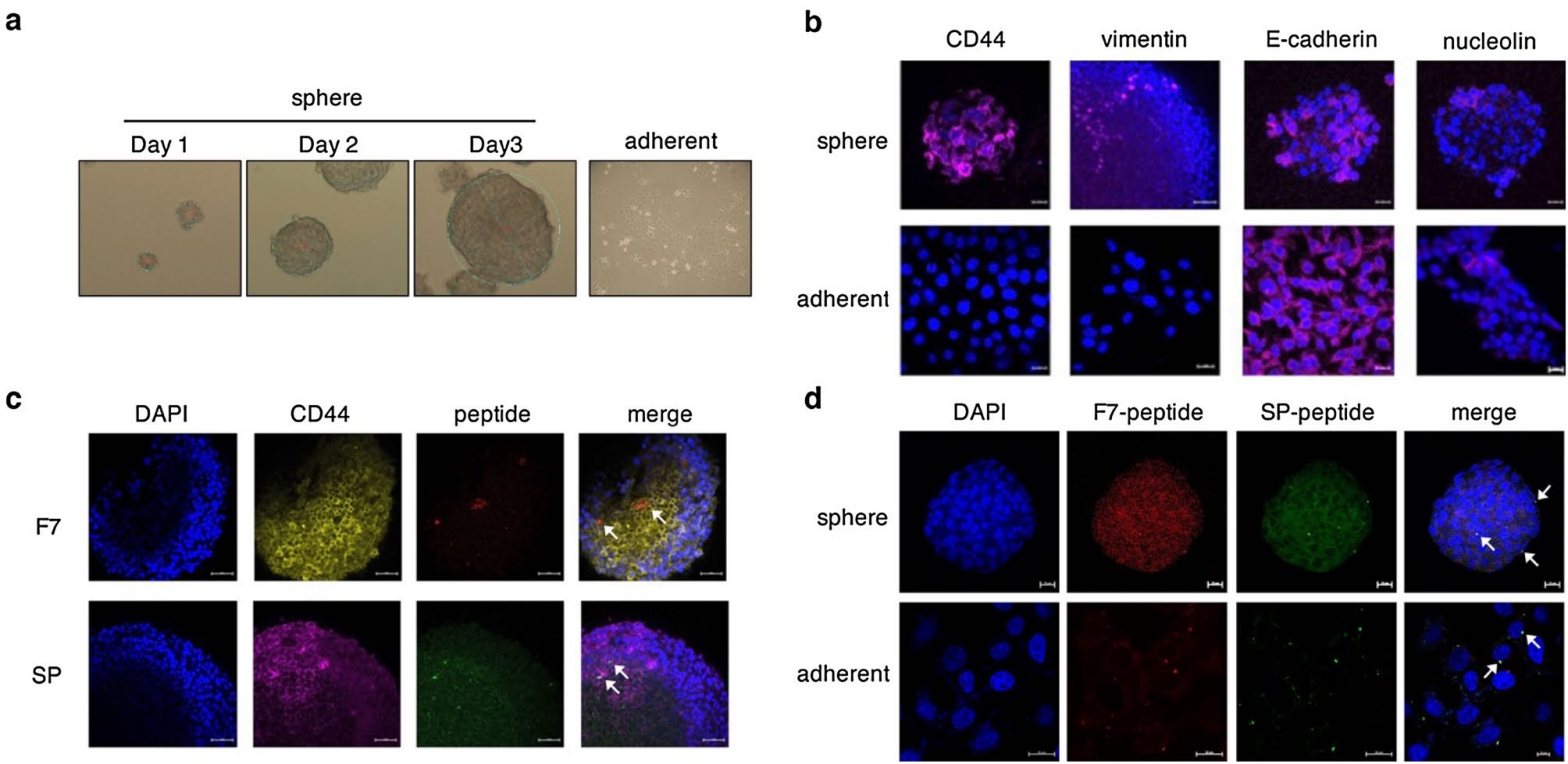
Expression of CSC markers corresponding to peptide binding. (**a**) Phase-contrast photomicrographs of the spheres cultured from SAS-E2-crimson-P2A::ttksr39 cell line using the ultra-low attachment plate. Right panel: adherent cells. magnification, 100×. (**b**) ICC results showing the expression of representative CSC marker, CD44 and EMT markers, vimentin and nucleolin in spheres (upper panel) and adherent cells (lower panel). E-cadherin is an epithelial cell marker. (**c**) The peptide binding to tumor spheres which highly expressed CD44. The arrow indicates the presence of F7 and SP peptide. Red: Lissamine Rhodamine labeled F7 peptide, Green: FITC labeled SP peptide, Blue: DAPI; Scale bar, 20 μm. (**d**) Comparison of peptides binding to spheres and adherent cells. Yellow: colocalization.

Studies have indicated that cultured tumor spheres exhibit the characteristics of CSCs including stemness, self-renewal and resistance to drugs and radiotherapy^14,15^. Thus, a non-adhesive culture system was used in this study to generate tumor spheroids of SAS cells for enrichment of CSCs cells or EMT-derived cells. The spheres were prepared and the morphological alteration was observed using optical microscope for 3 consecutive days (Fig. 2a). The spheres showed a round shape, smooth contour and clustered in a three-dimensional configuration growing gradually over time in comparison with adherent cells. To further investigate whether the cells undergo the process of EMT, the representative CSC/EMT markers CD44, E-cadherin, vimentin and nucleolin were used to differentiate the spheres from adherent cells. Immunostaining results showed the expressions of CD44 and vimentin increased in spheres, whereas the expression of E-cadherin was decreased (Fig. 2b). It is suggested that there was a remarkable increase in EMT after spheroid formation. Notably, the expression of nucleolin remained unchanged in spheres and adherent cells. After incubating with fluorescein-labeled peptides for 24 h, the red fluorescence and green fluorescence were observed in CD44 expressing spheres, suggesting that both F7 and SP peptides are able to bind and detect CSCs (Fig. 2c). Considering the tumor heterogeneity, both the spheres and adherent cells were incubated with fluorescein-labeled peptides respectively to evaluate the binding activity of peptides (Fig. 2d). The confocal images showed that F7 and SP peptides bound to cancer cells grown either in adherent or in spheroid form. Moreover, some of the two peptides were found to colocalize in cells (Fig. 2d, white arrows).

### In vitro induction of EMT and stemness with Unc0642/IR

**Figure 3:**
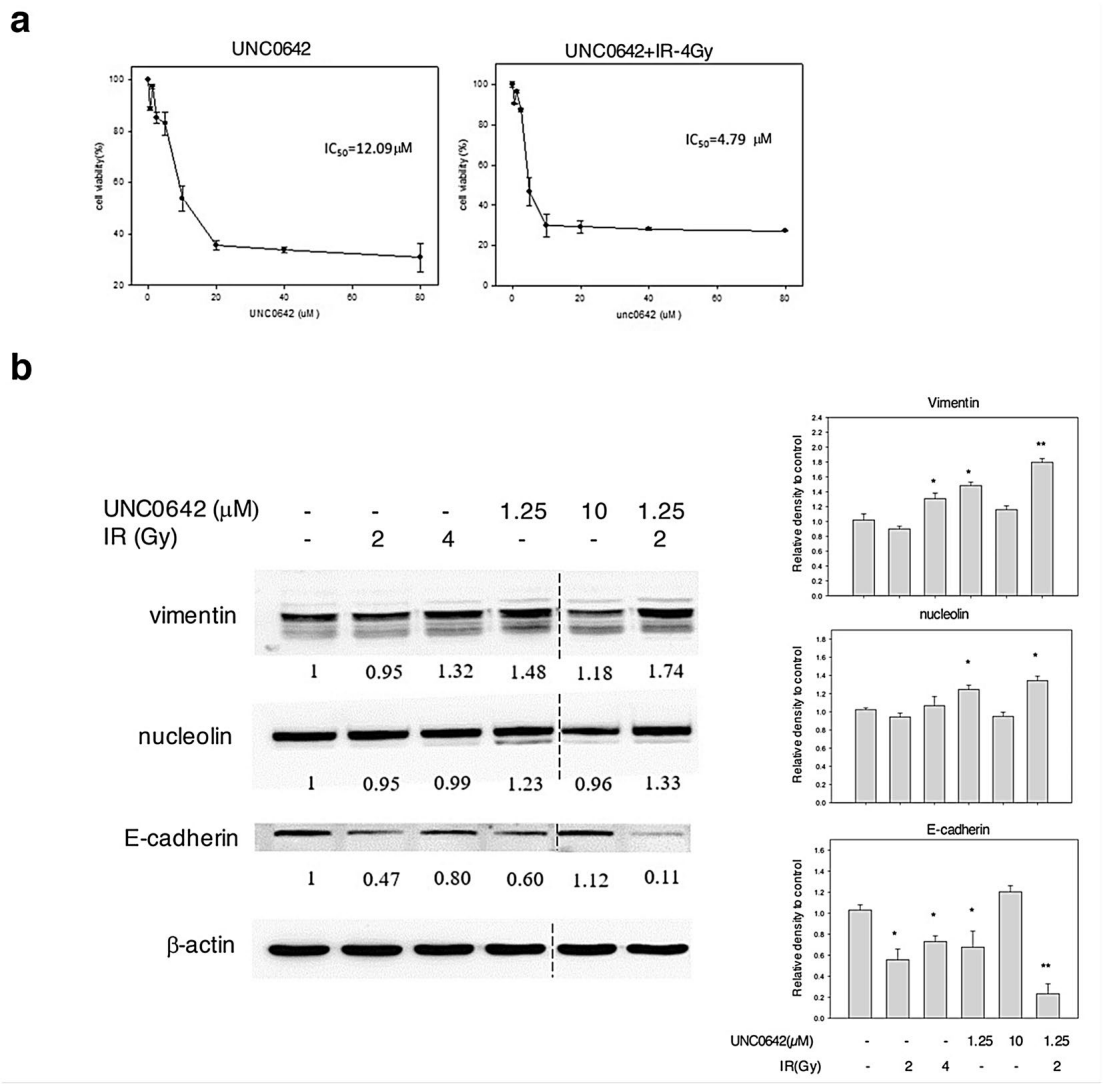
In vitro induction of EMT and stemness with Unc0642/IR. (**a**) SAS cells were treated with indicated concentration of Unc0642 and Unc0642 + 2 Gy of radiation for 72 h respectively. Cell viability was assessed by AlamarBlue assay. All data were normalized to cells without treatment. Data are presented as mean ± SD. IC_50_ was calculated using SigmaPlot12 software. (**b**) Western blot analysis of CSC and EMT markers in SAS cells treated with different concentration of Unc 0642 (1.25 and 10 µM) and different dose of radiation (2 and 4 Gy) respectively. Internal control: b-actin. Right graph: the quantification of protein expression levels normalized against b-actin levels in each sample. *p < 0.05; **p < 0.01 by Student’s *t*-test. The blots were cropped from different parts of the same gel. The original blots and all replicates are presented in Supplementary Fig. 4.

To increase the number of EMT-derived cells, Unc0642 and low-dose IR were used to simulate the cancer recurrence after chemotherapy and radiotherapy in our study. Small molecule inhibitor specifically targeting G9a (a lysine methyltransferase) has been found to disrupt DNA repair, thereby increasing sensitivity to radiation^16^. In this study, Unc0642 was chosen to inhibit G9a function. Accumulating evidence indicates that radiation can accelerate the spread of primary tumors and development of distant metastasis, attributing to the induction of EMT and the presence of CSCs with radioresistance^17,18^. At first, the IC_50_ (50% inhibition concentration) was determined for Unc0642 and combined treatment with low-dose IR (Fig. 3a). The IC_50_ value for SAS cells exposed with Unc0642 for 72 h was 12.09 μM. When combining with 4 Gy of IR, the IC_50_ was found to decrease and occur at 4.79 μM. Of note, there was a peak at a dose of 1.25 μM in either single treatment or combined treatment, indicating this dose of Unc0642 could induce cell proliferation. Transforming growth factor-β1 (TGF-β1), as a multifunctional cytokine, has been reported to enhance cell migration and invasion by inducing EMT^19^. Thus, we ensured that the treatment of TGF-β1 upregulated the expression of vimentin but downregulated the expression of E-cadherin, as judged by immunoblotting (Fig. S2). Next, the effects of Unc0642 and IR on the EMT on SAS cells were explored. As shown in Fig. 3b, the expression of vimentin was increased in SAS cells exposed to IR (2 and 4 Gy) and 1.25 μM Unc0642, whereas the level of vimentin was almost unchanged in cells treated with 10 μM Unc0642. SAS cells exposed to 2 Gy of IR and 1.25 μM Unc0642 showed ~ twofold higher vimentin expression compared to control SAS cells (*p* < 0.01). The level of nucleolin was slightly increased in the presence of 1.25 μM Unc0642 (*p* < 0.05). In addition, we found a diminished protein expression level of E-cadherin in SAS exposed to Unc0642 or IR alone. Corresponding to the critical steps in EMT, high vimentin expression and loss of E-cadherin expression were found in the combined Unc0642- and IR-treated SAS cells. These results showed that the combination of Unc0642 and radiation therapy could be an effective method to promote cancer stemness and EMT phenotype.

### Evaluation of peptide binding activity to EMT derived cells induced by Unc0642/IR

**Figure 4:**
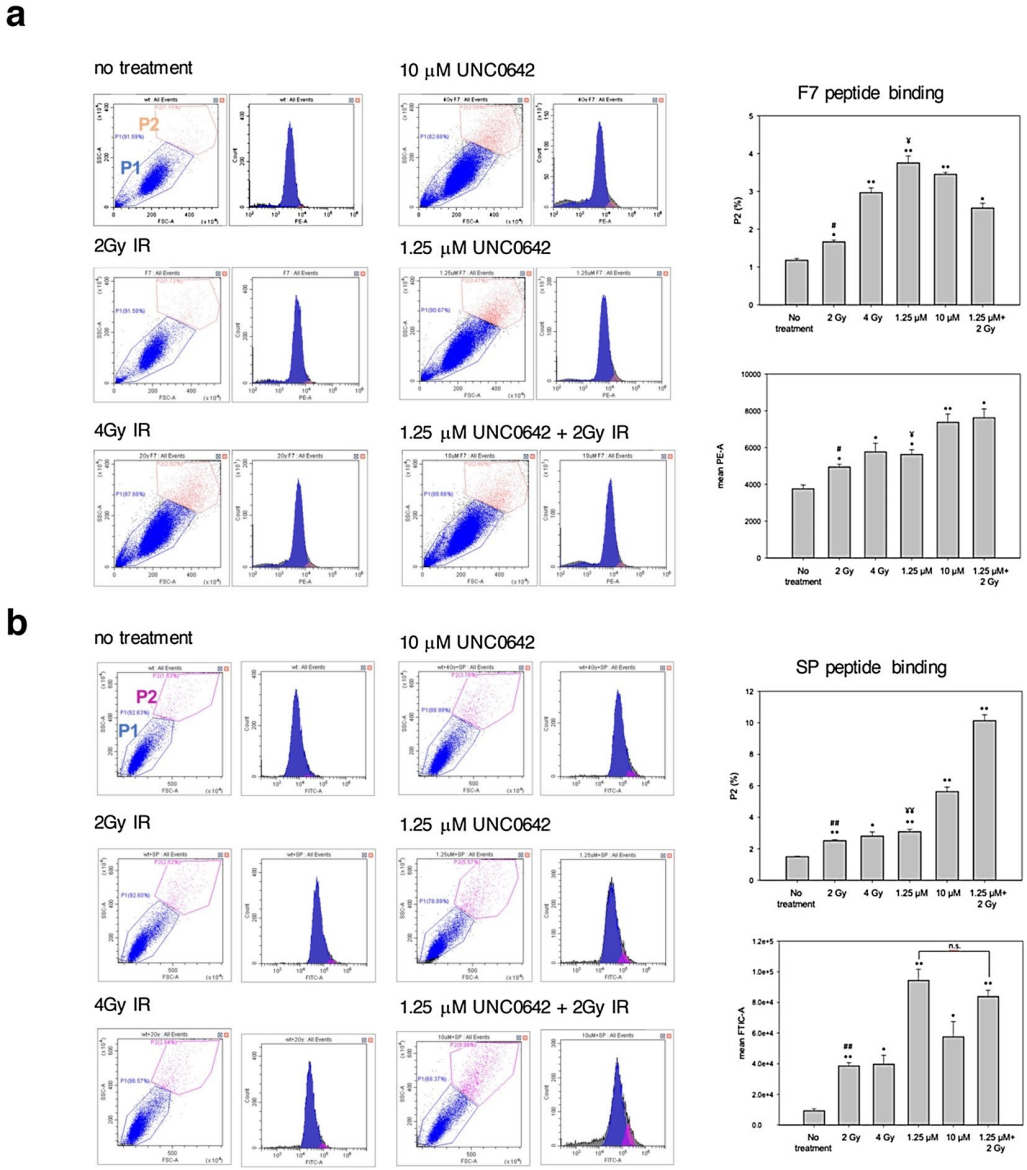
Evaluation of peptide binding activity to EMT derived cells induced by Unc0642/IR. After treatment of Unc0642 or radiation, EMT derived cells were incubated for 24 h in the presence of 50 ng/μl of fluorescein-labeled F7 (**a**) and SP peptide (**b**) at 37 °C respectively. FACS analysis was performed on gated live cells and light scatter parameters were measured using the 488 nm laser. The dot plot represented the particle size (FSC) versus granularity (SSC) of the cell population. The histogram represented the number of cells from P1 and P2 regions displaying a given fluorescence signal. Right graph: the quantitative analysis in the ratio of fluorescein-labeled peptides binding to SAS cells. Symbols (*) denotes significantly different from no treatment group, (^#^) and (^¥^) denote significantly different from 1.25 μM + 2 Gy group, respectively. *, ^#^, ^¥^*p* < 0.05, **, ^##^, ^¥¥^*p* < 0.01, n.s., nonsignificant by Student’s *t*-test.

To investigate whether the peptide binding is increased by the induction of EMT, fluorescein-labeled F7 and SP peptides were added into EMT-induced SAS cells for 24 h respectively and FACS analysis was used to monitor peptide binding. The population of F7 peptide-binding cells was increased with increasing dose of IR and Unc0642, reflected by the enhanced red fluorescence (Fig. 4a). Likewise, we found the SP peptide bound to EMT-induced SAS cells and particularly the average fluorescence intensity (FITC) was significantly higher in combined Unc0642- and IR-treated SAS cells (Fig. 4b). The results indicated the potential of F7 and SP peptides to detect EMT derived cells with high expression of vimentin and nucleolin.

### Evaluation of peptide binding ability in SAS xenograft mouse model after the treatment of Unc0642/IR

**Figure 5:**
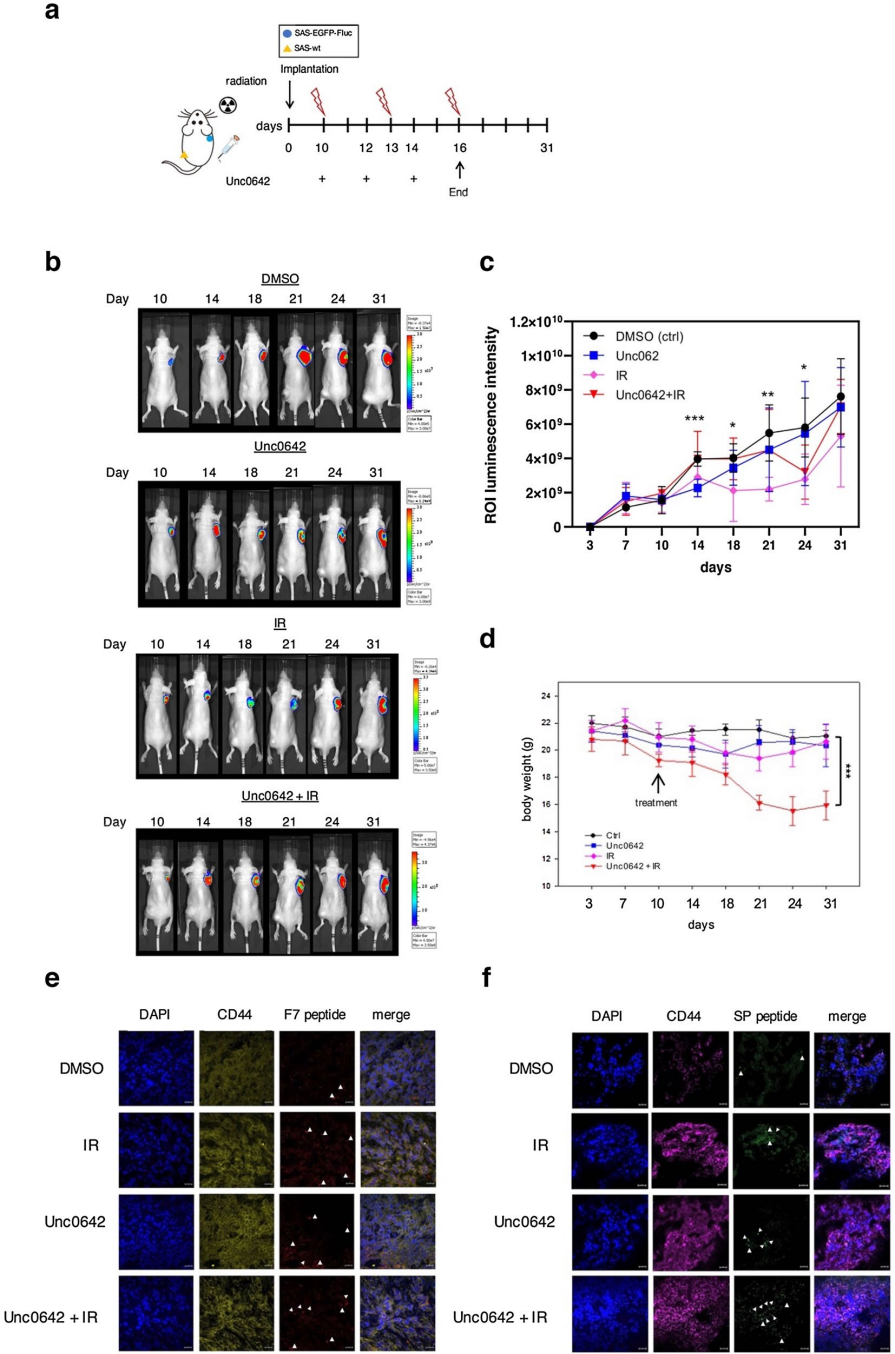
Evaluation of peptide binding ability in SAS xenograft mouse model after the treatment of Unc0642/IR. (**a**) Schematic diagram for in vivo EMT induction via the treatment of Unc0642 and radiation. (**b**) Evaluation of the effect of Unc0642/IR in SAS-EGFP-Fluc-bearing mice by optical bioluminescence image. (**c**) Tumor burden of each group was monitored at indicated time points. Data represent mean ± SD (n = 5–6/group). *p < 0.05, **p < 0.01, ***p < 0.001 by Student’s *t*-test. ***p = 0.00058, Ctrl versus Unc0642 on day 14; *p = 0.049, Ctrl versus IR on day 18; **p = 0.0023, Ctrl versus IR on day 21; *p = 0.0179, Ctrl versus IR on day 24; *p = 0.036, Ctrl versus Unc0642 + IR on day 24. (**d**) The body weights of mice were recorded during the period of monitoring. Data are mean ± SD (n = 5–6/group). (**e**, **f**) Mice were given an intra-tumoral injection of peptide. At 24 h later, tumor samples were excised and the signal derived from the peptide was observed using confocal microscopy. Representative confocal micrographs of tissue sections immunolabeled with CD44. The arrowheads indicate the presence of F7 and SP peptide. Blue: DAPI. Scale bar, 20 μm.

To investigate the binding ability in vivo, we established a xenograft mouse model by implanting SAS-EGFP-Fluc tumor cells into nude mice subcutaneously. When the tumors reached approximately 100 mm^3^, the mice received an intraperitoneal injection of Unc0642 followed by IR starting from day 10 to day 16; the procedure for in vivo EMT induction was as shown in Fig. 5a. Tumor growth was monitored by bioluminescence imaging (Fig. 5b). Mice treated with Unc0642 or IR alone showed a clear delay in tumor growth, the Fluc signal decreased in comparison with control group on day 18, indicating that growth inhibition effect persisted for at least 1 week. Interestingly, no significant reduction was observed in Unc0642 plus IR treated mice until Day 21, but drastically dropped down on day 24. The tumor burdens in the mice receiving the IR alone and combined treatment of Unc0642 and IR suddenly increased on day 31, whereas the tumor burden in untreated mice kept growing constantly (Fig. 5c). Body weights were recorded twice a week before and after different treatments (Fig. 5d). Initially, there was no difference between the mice treated with Unc0642/IR alone and those treated with DMSO (the control group). Drastic weight loss occurred only in mice receiving the combined treatment of Unc0642 and IR until the end point of the study (*p* < 0.001). In light of these results, the tumor xenograft mouse model treated with drug and radiation successfully exhibited the tumor relapse and a malignant tendency of cancer cells.

To evaluate whether these two peptides are able to detect EMT derived cells, the mice implanted with SAS-EGFP-Fluc cells were given an intra-tumoral injection of F7 peptide and the mice implanted with SAS-wt cells were given an intra-tumoral injection of SP peptide, respectively on day 31 (Fig. S3). After 24 h, analysis of peptide binding and EMT markers from the excised tumor tissue was performed by immunofluorescence (IF) staining. The tumors from Unc0642/IR alone or Unc0642 plus IR-treated groups displayed higher CD44 expression than that of the control group (Fig. 5e and f). The amounts of fluorescein labeled F7 peptide and SP peptide were also increased in tumor tissues respectively. These data were in accordance with the in vitro findings that F7 peptide and SP peptide have the potential to detect EMT derived cells.

## Discussions

Our results demonstrated that F7 and SP peptides are able to penetrate into cytoplasm of SAS cells specifically. In addition, in vitro and in vivo induction of EMT studies showed that these two peptides could detect EMT derived cells or CSCs with high nucleolin and vimentin expression.

In cancer research, approaches in identification and isolation of subpopulations within tumors are based on the expression pattern of cell surface markers. Although the selection of unique markers is the first priority, the subsequent detection and isolation methods are another big issue that is related to the efficiency and translation into clinical utility^20^. Targeting selected markers via magnetic beads conjugated antibodies or fluorescein labeled antibodies are common methods with high specificity. The use of peptides offers several advantages in comparison with antibodies or ligands, including (1) low molecular weight and poorly immunogenicity, (2) ability to penetrate through cell membranes, (3) ease of synthesis using phage display and low cost, and (4) simple formulation assembly. Thus, the availability of such specific peptides might be suitable for the improvement of the abovementioned methods. In this regard, F7 and SP peptides that specifically bind to CSC marker nucleolin and EMT mesenchymal marker vimentin respectively can serve as a connector in the isolation system, such as Biotin-Avidin system and FACS or magnetic‐activated cell sorting (MACS) techniques, to isolate the subpopulations with EMT/CSC properties. In addition, these peptides can be used in diagnostic or therapeutic applications, such as pharmaceutical carriers to selectively deliver imaging agents or chemotherapeutic agents^21^. For example, the liposomal doxorubicin conjugating F7 peptides specifically targets to nucleolin in MCF-7, a breast cancer cell line and accumulates within cells through enhanced permeability and retention effect (EPR effect), thereby reducing side effects and dose-limiting toxicities^22,23^.

Despite the cancer cells captured by F7 or SP peptide were identified as CSCs that expressed CD44, single peptide binding cells (F7+ or SP+) and double peptides binding cells (F7+ /SP+ , as shown in Fig. 2d) were considered as different subpopulations that exist at the different stages of cancer. Additionally, we found that the binding patterns were inconsistent when F7 and SP peptide bound to EMT derived cells (Fig. 4) and the signals representing F7 and SP peptides were partially overlapped with CD44 (Fig. 5e and f). There are several possibilities for these findings. First, the permeability, stability and retention of these peptides in vivo might be taken into consideration. In the described experiments, the mice were given an intra-tumoral injection of F7 and SP peptide respectively 24 h before the IHC analysis of excised tumor samples. These peptides have a high stability at 37 °C in the presence of serum according to previous studies and our results also showed that the F7 and SP signals could be still found when incubation with SAS cells for 24 h (Fig. 1b and c). However, the tumor microenvironment, including the surrounding blood vessels, immune cells and fibroblasts, may contribute to the reduction of peptides. Second, other CSC markers might be required for evaluation of peptide binding owing to the existence of various subpopulations. Ghuwalewala et al. reported that the subpopulations isolated from a set of OSCC cell lines can be re-defined using CD44 and CD24 markers along with subsequent molecular and cellular profiles^24^. CD44^high^CD24^low^ cells have been demonstrated to have increased CSC and EMT properties while CD44^low^CD24^high^ cells are regarded as non-stem cells. Notably, CD44^high^CD24^high^ cells exhibit traits which are transitional between the non-stem and the stem-like, representing an intermediate population. In addition to CD44, other CSC markers might be used to reflect the tumor heterogeneity and meanwhile to evaluate the peptide binding.

In the current study, we successfully established a protocol for EMT induction via the combination of the G9a inhibitor and low-dose IR. G9a, mainly catalyzes histone H3K9 methylation, is shown to be overexpressed in many types of cancers, including gastric cancer, ovarian cancer, head and neck cancer, etc. Higher G9a expression levels represent higher methylation levels, thus leading to the transcriptional repression of tumor-suppressor genes and are also associated with poor prognosis in patients^25^. Liu et al. explored that snail-induced EMT and repression of E-cadherin in head and neck squamous cell carcinoma requires the G9a protein^26^. G9a is also noted to be implicated in mediating the DNA repair through efficient homologous recombination, which make cancer cells more resistant to radiation and chemotherapies; therefore, inhibition of G9a catalytic activity to interfere DNA repair pathways is a rational therapeutic strategy against cancers^27^. Unc0642, as a selective G9a inhibitor, has been optimized to exhibit lower cell cytotoxicity, higher selectivity and improved in vivo pharmacokinetic properties, making it suitable for animal studies^28^. Cao et al. reported that the treatment with UNC0642 decreased the level of histone H3K9me2 and increased the expression of BIM, a proapoptotic protein, in urinary bladder cancer (UBC) cells. Thereby, apoptosis was induced and leaded to the suppression of tumor growth in UBC xenograft model^29^. Of note, there were no significant weight changes between the vehicle-treated group and UNC0642 treatment groups (5 mg/kg body weight, used in their study) at any time point post-treatment, indicating that the dose of UNC0642 used in vivo was effective and safe. In our study, the half dose was administered in mice based on the purpose of EMT induction rather than tumor eradication. Radiotherapy is widely used prior to surgery and other therapeutic strategies. IR induces single strand breaks or double strand breaks, which trigger the activation of DNA damage response signaling pathways to arrest cell-cycle progression and activate appropriate DNA repair mechanisms^30^. Previous findings indicate that IR can promote EMT mediated metastasis and gain of CSC phenotypes by TGF-β signaling^17,18,31,32^. In our mouse model, significant reduction of Fluc signal was observed after Unc0642/IR alone or Unc0642 plus IR treatments in the mice as compared with the mice treated with the same amount of DMSO (Fig. 5b and c). However, the tumor burdens in these treated mice suddenly increased on day 31 almost near that in control group, representing the occurrence of relapse. The results also demonstrated that Unc0642 and IR induced the EMT phenotype, decreased E-cadherin and increased vimentin expressions as well as CSC markers, CD44 (Figs. 3b and 5e, f). According to the results of our study, we would recommend that 1.25 μM Unc0642 plus 2 Gy of IR is the suitable dosage for the occurrence of EMT in SAS cells.

Although our results showed the potential of the detection approach for EMT derived cells or CSCs by using specific peptides, there were also several limitations. First, in vivo evaluation of peptide binding ability was carried out by intra-tumoral injection rather than systemic delivery, suggesting that certain subpopulations like circulating tumor cells (CTCs) were ignored. Since CTCs with CSC-like phenotype transport via the blood stream to distant sites and thereby form metastatic colonies, it is necessary to further investigate whether this detection approach using specific peptides can track CTCs. Second, in the current study, F7 and SP peptide were labeled with fluorescein followed by confocal microscopic imaging and FACS analysis for the evaluation of peptide binding activity. However, more detailed information regarding the binding of F7 and SP peptides to nucleolin and vimentin respectively is required for the potential application of these tumor targeting peptides in detection and isolation approaches. For instance, the isothermal calorimetry (ITC) is a widely used label-free quantitatively measurement of the binding affinity and thermodynamics for biomolecular interactions, such as protein–protein, protein-small molecules or protein-DNA, might enable us to understand functions and mechanisms at a molecular level^33^. Third, whether the peptide binding to its target expressed in cancer cells causes any cellular alteration by activating the signaling pathway pertaining to angiogenesis, tumorigenesis or apoptosis is our concern. In fact, SP peptide targeting to vimentin was shown to induce angiogenesis under hypoxic conditions^13^. It might be a potential risk of tumorigenesis if applied for in vivo molecular imaging and targeted therapeutic delivery. Thus, evaluations of the course of treatment, administered dose and tumor microenvironments are warranted.

In summary, our study has provided in vitro and in vivo evidences suggesting that the F7 and SP peptides could penetrate into cell cytoplasm and detected EMT derived cells and CSCs with high nucleolin and vimentin expression. Furthermore, the binding of these peptides was increased after the induction of EMT by the combination of small molecule inhibitor and low-dose IR. Our study suggests that these peptides may serve as a tool to detect EMT derived cells or CSCs and might aid the development of isolation methods for understanding these subpopulations within tumors in cancer research.

## Methods

### Cell culture

The human tongue squamous carcinoma derived cell line, SAS was obtained from the ATCC (a kind gift from Dr. Muh-Hwa Yang) and HEK293, a human kidney fibroblasts cell line, was provided from Dr. Yi-Jang Lee (Department of Biomedical Imaging and Radiological Sciences, National Yang Ming Chiao Tung University, Taipei, Taiwan). Cells were cultured in Dulbecco’s modified Eagle’s medium (Thermo Fisher Scientific, Inc., Waltham, MA, USA) supplemented with 10% fetal bovine serum (Biological industries, Israel) and 1% penicillin–streptomycin in a 5% CO_2_ humidified incubator at 37 °C.

### Generation of stable cell lines

To generate SAS cells stably expressing dual reporter genes EGFP-Fluc or E2-crimson-P2A::ttksr39, cells were transduced with lentiviral vector for 6 h and then replaced with regular growth medium. Two days after the transduction, the cells were replated in 100 mm^2^ culture dishes with continually grown in the presence of 500 μg/mL G418 (Sigma-Aldrich) for approximately 2 weeks. G418 resistant colonies were isolated and cultured in G418 containing media. The cell suspensions were filtered through 70 μm strainers and then sorted on a FACSAria system (Becton–Dickinson). CellQuest (Becton–Dickinson) and FlowJo software (TreeStar) were used for analysis.

### In vitro induction of EMT

To induce the EMT of SAS cells, cells were seeded in 60 mm^2^ dishes at the density of 1 × 10^6^ cells/dish overnight and incubated with 1.25 and 10 μM Unc0642. After 24 h, cells were exposed under 2 Gy and 4 Gy of radiation by a RS2000 Series Biological Irradiator (Rad Source Technologies Inc, Buford, GA, USA) and replaced with fresh Unc0642 containing medium every 24 h. The expression of vimentin and E-cadherin were detected by western blotting.

### Peptide synthesis

The SP peptide (SAHGTSTGVPWP) and F7 peptide (DMPGTVLP) were purchased from GenDiscovey Biotechnology Inc. (Taipei, Taiwan). The SP peptide was synthesized with the addition of N-terminal fluorescein isothiocyanate (FITC) and the F7 peptide were labeled with Lissamine Rhodamine. The purity of all of the peptides was greater than 95%.

### Fluorescence-based peptide binding assay

SAS-EGFP-Fluc and SAS-E2-crimson-P2A::ttksr39 were seeded on 8-well chamber slides and incubated with fluorescein labeled F7 peptide and SP peptide respectively for the different time points (0, 0.25, 0.5, 1, 2, 4, 8, 12 and 24 h). HEK293 cell lines without expression of vimentin and nucleolin served as a negative control for the specificity of peptides. After removal of the medium and a PBS rinse, the cells were fixed with 3.7% paraformaldehyde for 10 min followed by PBS washing for 3 times. Subsequently, all slides were mounted with VECTASHIELD Mounting Medium with DAPI (cat. H-1200, Vector Labs, Burligame, CA, USA) and covered with cover glasses before visualization under a laser-scanning microscope (Zeiss LSM 880, Jena, Germany) with Zen Blue software (ZEISS, Jena, Germany).

### Flow cytometry analysis of peptide binding

The cells were treated for EMT induction as described above. Then, 50 ng/μl of F7 and SP peptides were added into the culture medium and co-incubated for 24 h. The cells were detached and fixed with 3.7% paraformaldehyde for 15 min followed by resuspension in PBS. FACS analysis was performed on gated live cells using a flow cytometer (Beckman Coulter CytoFLEX, Brea, CA, USA).

### Western blotting

Cultured cells were placed on ice, washed by ice-cold PBS and lysed using CytoBuster Protein Extraction Reagent (cat. 71,009, Bedford, MA, USA) containing Protease Inhibitor Cocktail Set I (cat. 539131, Millipore, Bedford, MA, USA). Cells were then scraped, collected in Eppendorf tubes and the sonicated for 30 min. The lysate was spun down at 12,000 g for 10 min at 4 °C. The protein content was measured using Bradford reagent (BIO-RAD, Hercules, CA, USA). The equal amounts of protein (40 μg) were separated in SDS-PAGE gels (30% Acrylamide/Bis Solution, 29:1, cat. 1610157, BIO-RAD, Hercules, CA, USA) and transferred to PVDF membranes (cat. NEF1002001PK, Perkin-Elmer life sciences, Boston, MA, USA). For immunoblotting, membranes were cut in terms of the molecular weight range prior to hybridization with primary antibody for vimentin (GTX100619, GeneTex, Irvine, CA, USA), nucleolin (GTX132778, GeneTex, Irvine, CA, USA), E-cadherin (GTX50757, GeneTex, Irvine, CA, USA) and b-actin (GTX109639, GeneTex, Irvine, CA, USA) overnight at 4 °C and then incubated with horseradish peroxidase HRP-conjugate goat anti-rabbit IgG (GeneTex, Irvine, CA, USA). Protein signals were visualized using a LumiFast Chemiluminescence Detection Kit (cat. JT96-K001S, T-Pro Biotechnology, Taiwan). Image capture was performed on an ImageQuant LAS4000 biomolecular image (GE healthcare life, Chicago, IL, USA). The intensities of protein bands were quantitated using ImageJ Gel Analysis program. Quantification of protein expression was performed by Image J. b-actin was used as loading control.

### Cancer spheroid culture and immunocytochemistry (ICC)

SAS cells were seeded in a 6-well cell culture plate with ultra-low attachment (Corning Costar, Tewksbury, MA, USA) at the density of 1 × 10^5^ cells/well and incubated for 3–5 days in a 5% CO_2_ humidified incubator at 37 °C. All measurements on the SAS spheroids were carried out with a diameter of approximately 0.75 mm. For ICC-IF staining, the spheroids were fixed in 3.7% paraformaldehyde, washed with PBS and permeated with 0.1% Triton X-100 for 10 min before blocking with 20% FBS for 10 min at 37 °C, followed by the incubation with anti-CD44 antibody (GTX102111, GeneTex, Irvine, CA, USA) at 4˚C overnight. The IF staining was performed as described below.

### Immunofluorescence (IF) staining

Frozen tissues sections or cell culture slides were fixed in 4% paraformaldehyde, washed with PBS and permeated with 0.1% Triton X-100 for 5 min before blocking with 20% FBS for 10 min at 37 °C, followed by the incubation with anti-vimentin antibody (cat. MAB3400, Millipore, Bedford, MA, USA) at 4˚C overnight. Sections were washed three times in PBS, each wash lasting 5 min then stained with Cy5-conjugated goat anti- mouse IgG (1:1000, cat. ab6563, Abcam, Cambridge, UK) for 1 h in the dark. All slides were mounted with VECTASHIELD Mounting Medium with DAPI (cat. H-1200, Vector Labs, Burligame, CA, USA) and covered with cover glasses before visualization under a laser-scanning confocal microscope (Zeiss LSM 880, Jena, Germany) with Zen Blue software (ZEISS, Jena, Germany).

### Animal model

All animal procedures were performed in accordance with the Guide for Care and Use of Laboratory Animals and National Yang-Ming University’s IACUC number was 1080110. Immunodeficient male mice (BALB/cAnNCrj-nu/nu, six weeks old, 20 gm) were provided by National Laboratory Animal Center (NLAC), NARLabs, Taiwan. Mice were group-housed (6 animals per cage) in individually ventilated cages (IVC) systems and had unlimited access to food and water. The room was maintained at a temperature of 20 to 21 °C, relative humidity of 50% to 70% and had a 12-h light/dark cycle. For establishment of xenografts, 2 × 10^6^ SAS-EGFP-Fluc cells in 100 μL phosphate-buffered saline (PBS) were injected subcutaneously into the animal's right shoulder and 2 × 10^6^ SAS-wt cells were injected into the left leg. Growth of the implanted tumors was monitored twice a week and tumor volumes were estimated from caliper measurements according to the following formula: tumor volume = (lengthÍwidth^2^) Í0.523. When tumor size reached approximately 10 mm^3^, the mice were randomized into four groups of five animals per group for the following treatments: (1) DMSO (1 ml/kg body weight), (2) Unc0642 (2.5 mg/kg body weight), (3) 2 Gy of radiation, (4) Unc0642 (2.5 mg/kg body weight) and 2 Gy of radiation. Mice were given Unc0642 (cat. SML1037, Sigma-Aldrich, St. Louis, MO) intraperitoneally (i.p) every two days for 3 times. Mice were placed under a RS2000 Series Biological Irradiator and then exposed to 2 Gy of radiation every two days for 3 times. Additionally, all methods were performed in accordance with the relevant guidelines and regulations and the study was carried out in compliance with the ARRIVE guidelines.

### In vivo bioluminescence image

Tumor-bearing mice were anaesthetized with 2% isoflurane and then injected D-luciferin (150 mg/kg body weight diluted in PBS) into the mouse peritoneum. Fifteen minutes later, mice were placed in the imaging chamber and photo counts were acquired for 30 s by a bioluminescence imaging system (Perkin Elmer, Waltham, MA, USA). The luminescent intensity at tumor sites was analyzed using living image software 3.2 (IVIS 50 Imaging System, Perkin Elmer, Waltham, MA, USA). Bioluminescent signal was recorded as mean photons/s/centimeter^2^/steradian (photon/s/cm^2^/sr).

### Statistical analysis

SigmaPlot12 (Systat Software Inc., San Jose, CA, USA) was used to perform statistical analysis. Student’s *t*-test analysis was performed for normally distributed data. Results were presented as the average ± SEM. A *p* value of less than 0.05 was considered statistically significant.

### Ethics declarations

The animal experiment was conducted according to the Guide for Care and Use of Laboratory Animals, and approved by the Committee on the Ethics of Animal Experiments of the National Yang Ming Chiao Tung University (IACUC number: 1080110, permission date: 23 January 2019).

## Supplementary Information

Supplementary Information.

## Data Availability

The data presented in this study are available on request from corresponding authors.
