# Supplementary material for: Detection of cancer stem cells by EMT-specific biomarker-based peptide ligands
Source: Sci Rep. 2021 Nov 17;11:22430. doi: 10.1038/s41598-021-01138-0 (PMC8599855; doi:10.1038/s41598-021-01138-0)
Supplement: Supplementary file 1 — Supplementary Information. [file 41598_2021_1138_MOESM1_ESM.docx]

Detection of Cancer Stem Cells by EMT-Specific Biomarker-based Peptide Ligands

Yi-An Chen^1, 3^, Cheau-Ling Ho^3, 4^, Min-Tzu Ku^4, 5^, Luen Hwu^3, 4^, Cheng-Hsiu Lu^6, 7, 8^, Sain-Jhih Chiu^3^, Wen-Yi Chang^5^ and Ren-Shyan Liu^1, 2, 3, 4, 6, *^

^1^Institute of Clinical Medicine, National Yang Ming Chiao Tung University, Taipei 112, Taiwan

^2^Department of Nuclear Medicine, Cheng Hsin General Hospital, Taipei 112, Taiwan

^3^Molecular and Genetic Imaging Core/Taiwan Mouse Clinic, National Comprehensive Mouse Phenotyping and Drug Testing Center, Taipei 112, Taiwan

^4^Department of Biomedical Imaging and Radiological Sciences, National Yang Ming Chiao Tung University, Taipei 112, Taiwan

^5^Department of Nuclear Medicine, Taipei Veterans General Hospital, Taipei 112, Taiwan

^6^Industrial Ph.D Program of Biomedical Science and Engineering, National Yang Ming Chiao Tung University, Taipei 112, Taiwan

^7^Core Laboratory for Phenomics and Diagnostics, Kaohsiung Chang Gung Memorial Hospital, Kaohsiung 833, Taiwan

^8^Department of Medical Research, Kaohsiung Chang Gung Memorial Hospital, Kaohsiung 833, Taiwan

* Corresponding author: Prof. Ren-Shyan Liu, M.D., e-mail: rsliu@vghtpe.gov.tw.

**Figure S1**. Establishment of SAS cell lines with stable expression of fluorescence and luciferase. (a) Immunofluorescence images of stable cell clones, SAS-EGFP-Fluc and SAS-pCMV-E2-crimson-P2A::ttksr39 showing expression of EGFP and E2-crimson fluorescent proteins respectively. Blue: DAPI; BF, bright field. Scale bar, 20 μm. (b) Histogram plots showing the percentage of EGFP positivity in stable SAS-EGFP-Fluc and the percentage of E2-crimson positivity in stable SAS-pCMV-E2-crimson-P2A::ttksr39 compared to SAS-wt cells as analyzed by flow cytometry. (c) Comparison of luciferase activity between SAS-wt and SAS-EGFP-Fluc cells. (d) Growth curves of 3 different SAS cell lines. The doubling time of SAS-wt, SAS-EGFP-Fluc, SAS-pCMV-E2crimson-P2A::ttksr39 is 22.23, 22.59, 21.86 hours respectively. The data are represented as mean ± SD from 3 independent experiments.

**
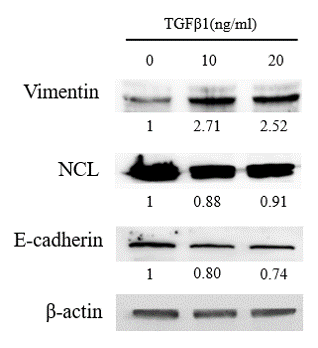
**

**Figure S2**. **In vitro induction of EMT and stemness with TGF-β1.** Western blot analysis of CSC and EMT markers in SAS cells treated with TGFβ1 (10 and 20 ng/ml). Internal control: β-actin. The quantification of protein expression levels was normalized against β-actin levels in each sample. The original and all replicates are presented in Figure S5.

**Figure S3**. SAS-wt tumor burden of each group was monitored at indicated time points.

**
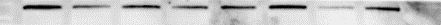

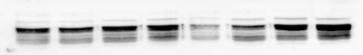

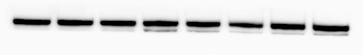

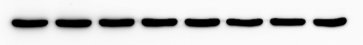
**

60

50

UNC0642 (μM)

IR (Gy)

-

-

5

-

-

4

1.25

-

-

2

5

2

10

-

1.25

2

100

**vimentin**

**nucleolin**

**E-cadherin**

**β-actin**

100

40

50

Replicate

**
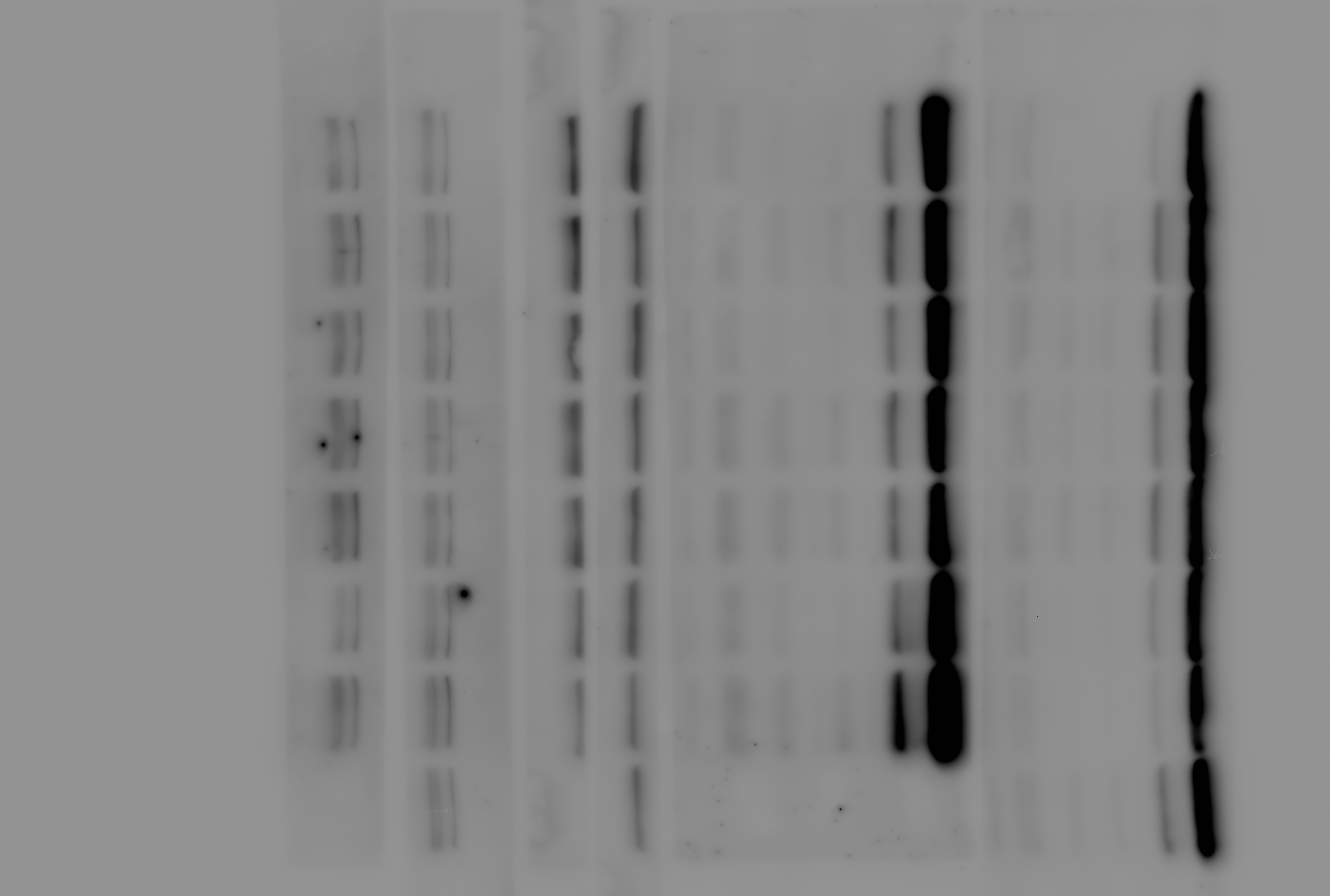

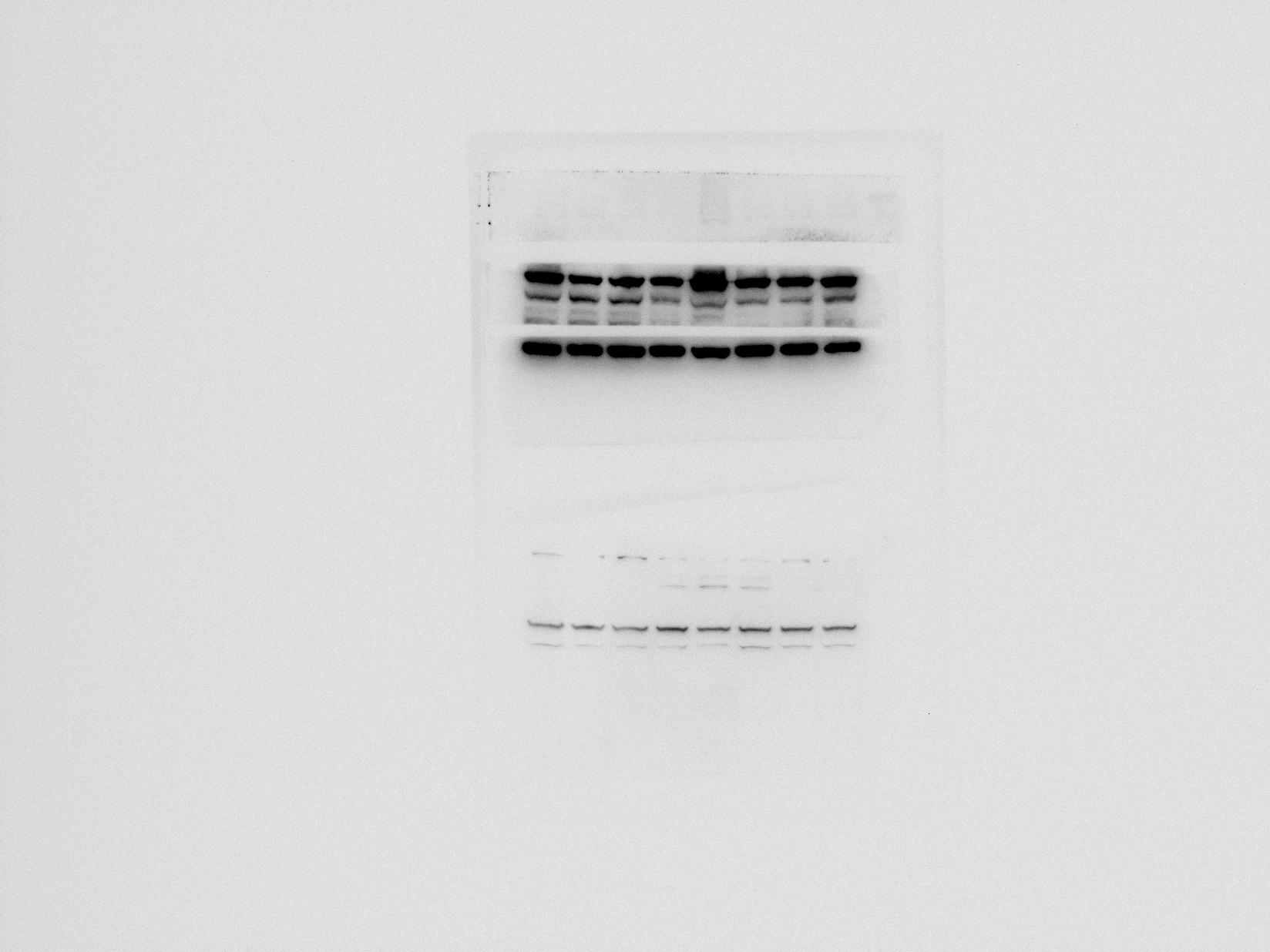

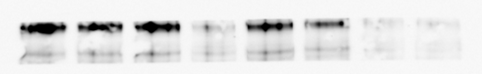

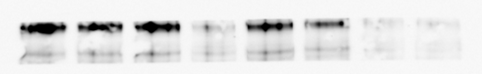

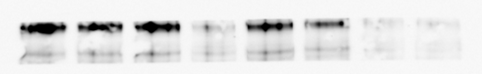

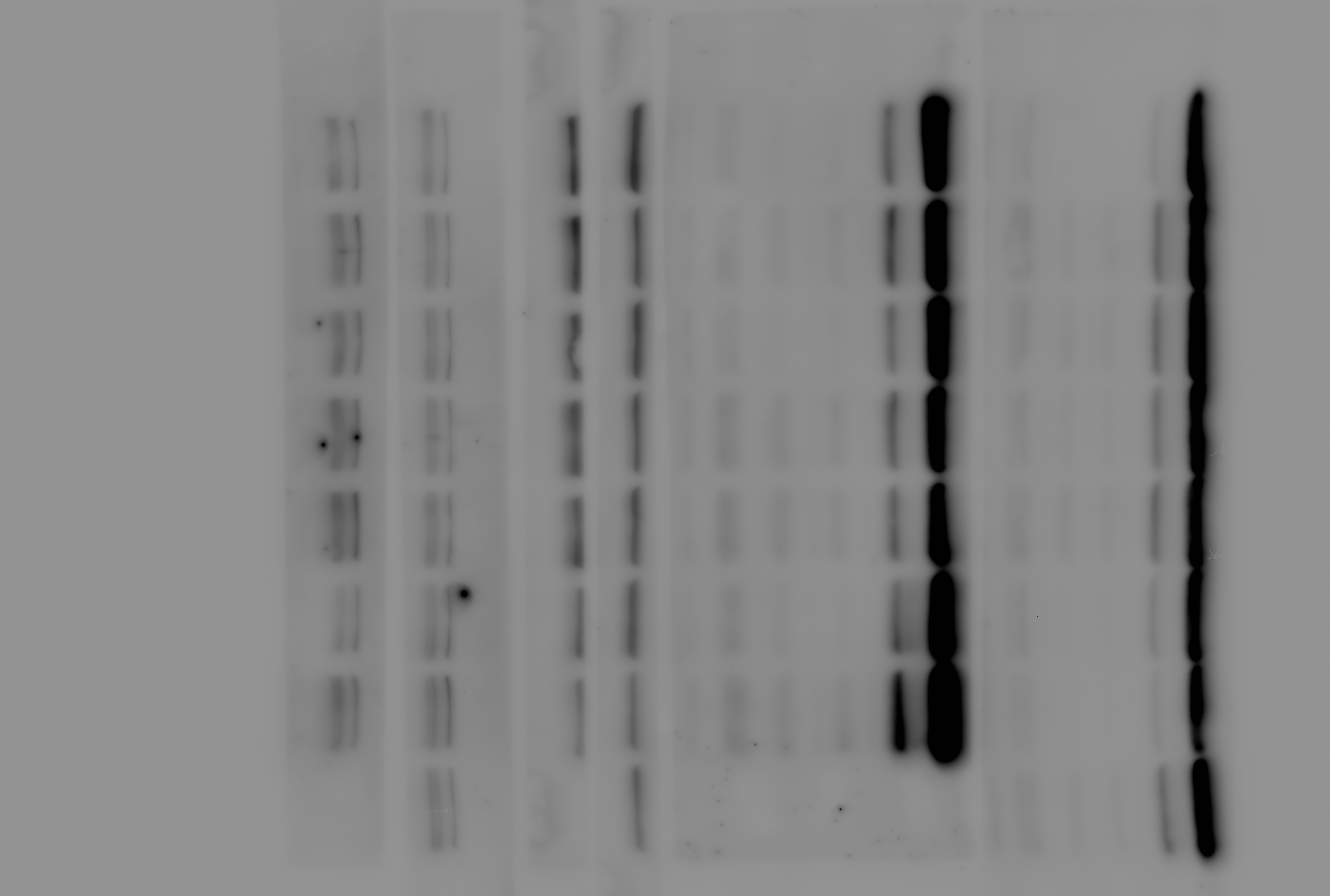
**

UNC0642 (μM)

IR (Gy)

-

-

5

-

-

4

1.25

-

-

2

10

-

1.25

2

**nucleolin**

**vimentin**

**β-actin**

**E-cadherin**

Replicate

**




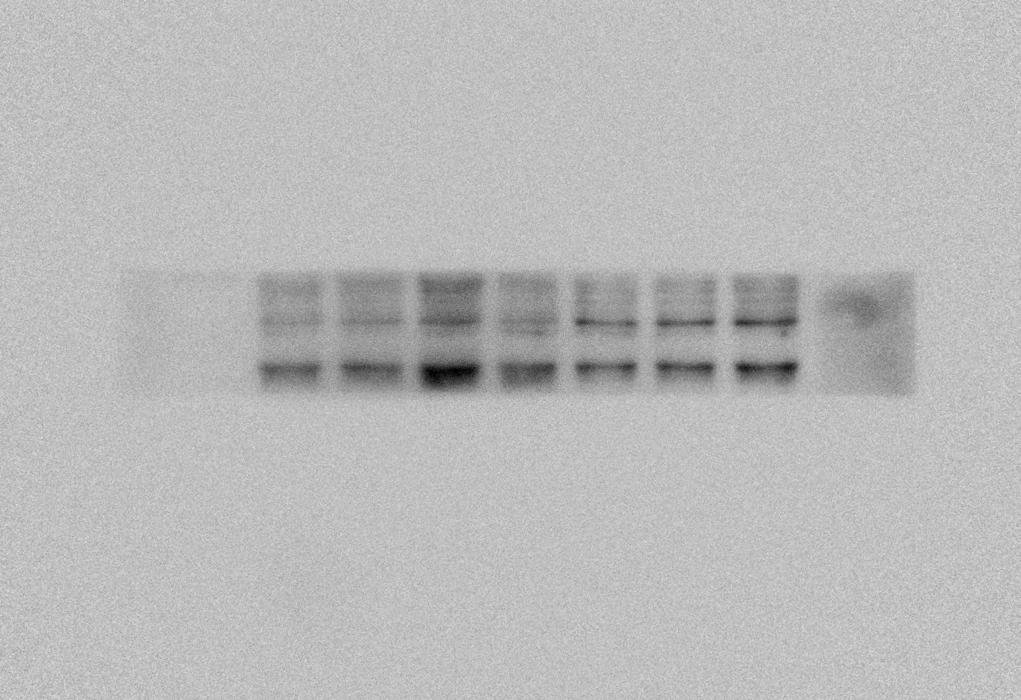

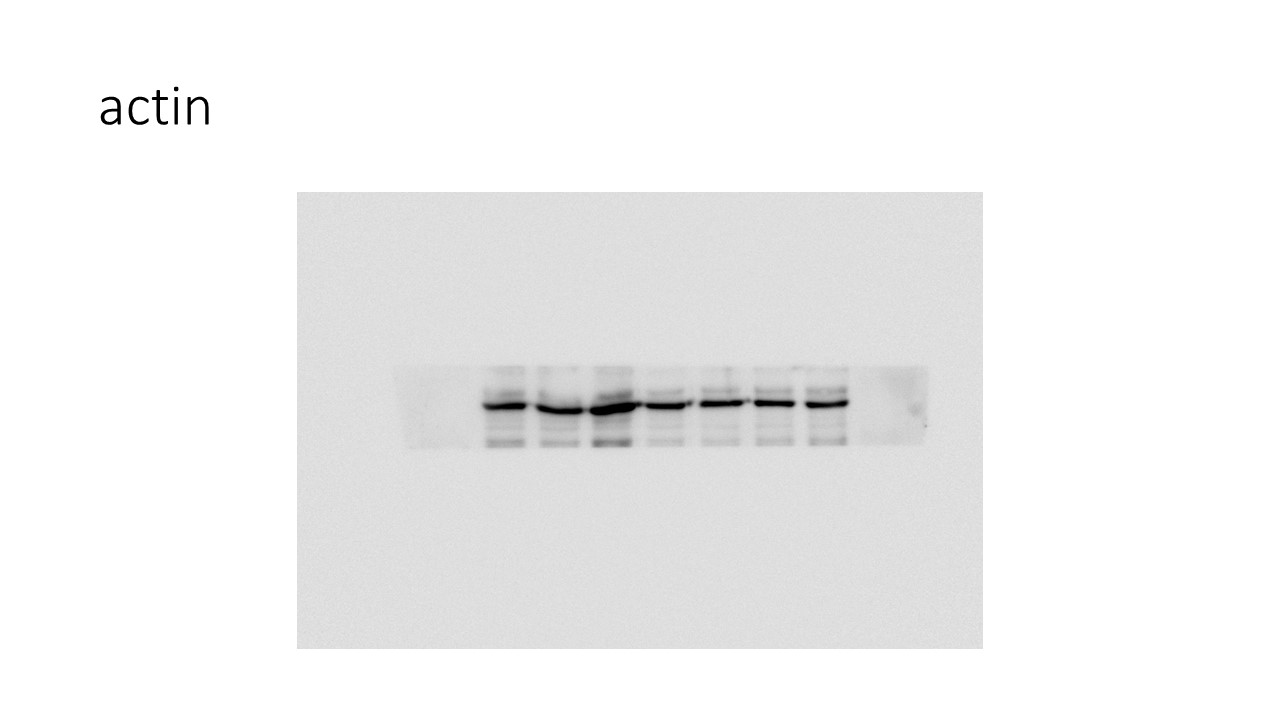
**

**β-actin**

**vimentin**

**nucleolin**

**E-cadherin**

UNC0642 (μM)

IR (Gy)

-

-

5

-

-

4

1.25

-

-

2

10

-

1.25

2

100

**Figure S4**. The full-length blots of Figure 3b and all replicates. For immunoblotting, membranes were cut in terms of the molecular weight range prior to hybridization with primary antibody.

0

10

20

TGFβ1 (ng/ml)

**
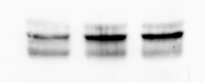
**

Vimentin

**
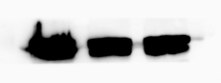
**

NCL

**
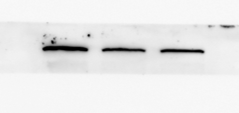
**

E-cadherin

**
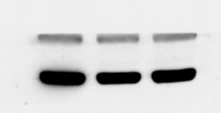
**

β-actin

Replicate

TGFβ1 (ng/ml)

20

10

0

**
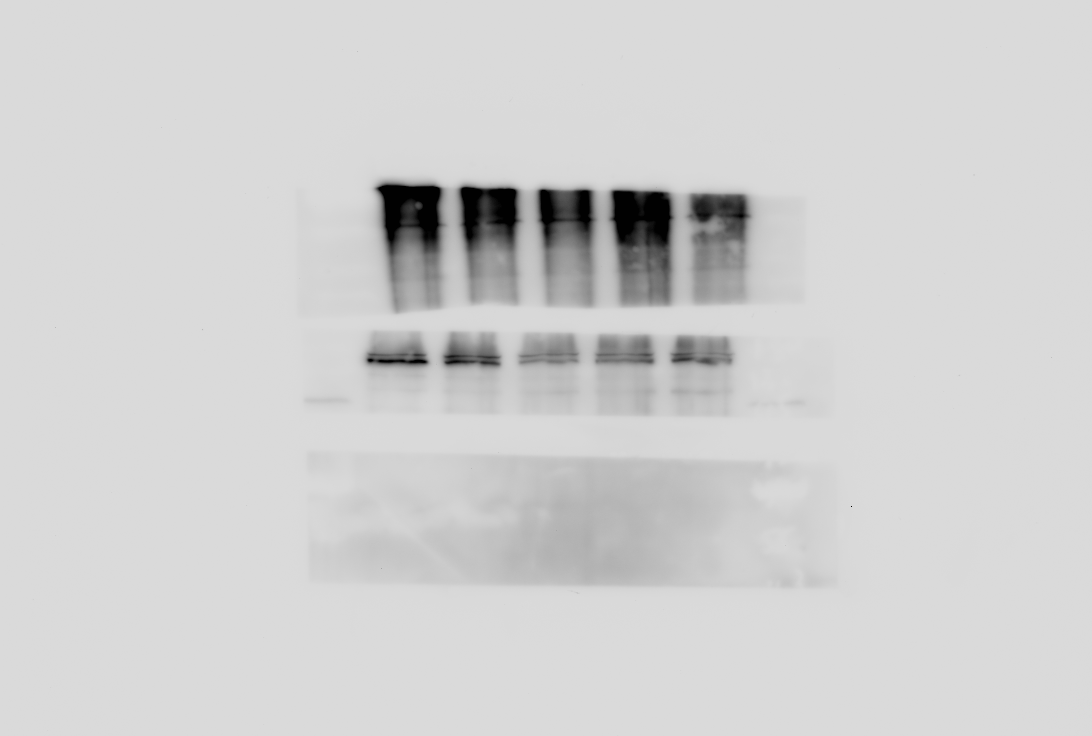
**

Vimentin

**
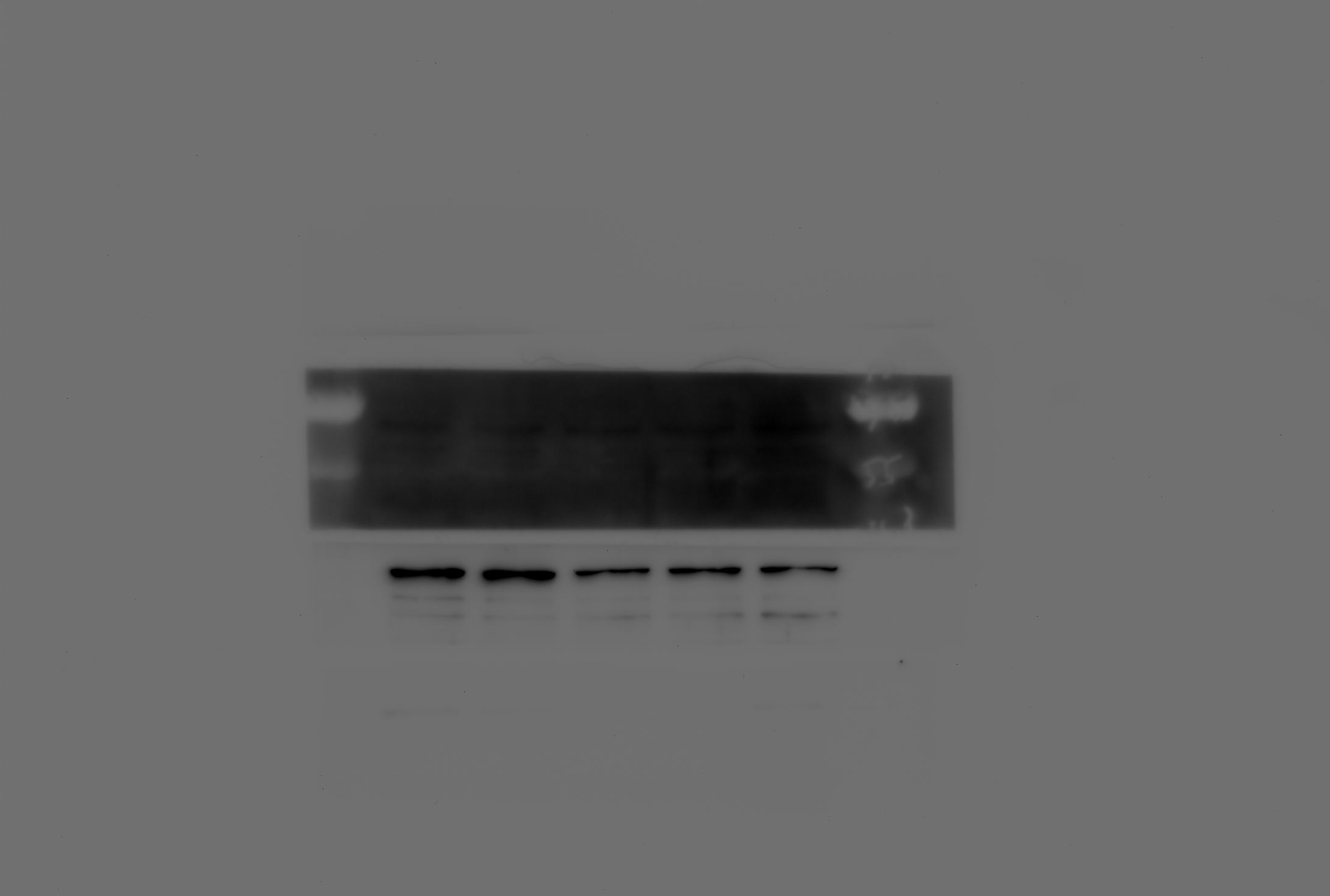
**

NCL

**
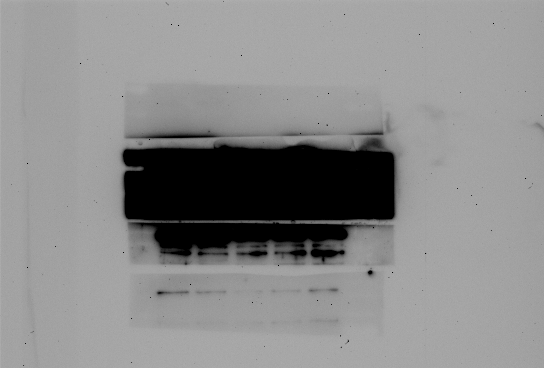
**

E-cadherin

**
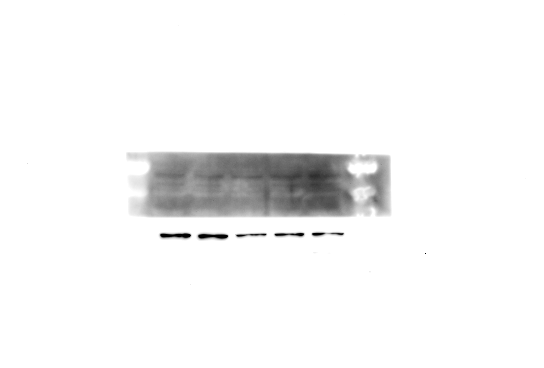
**

β-actin

**
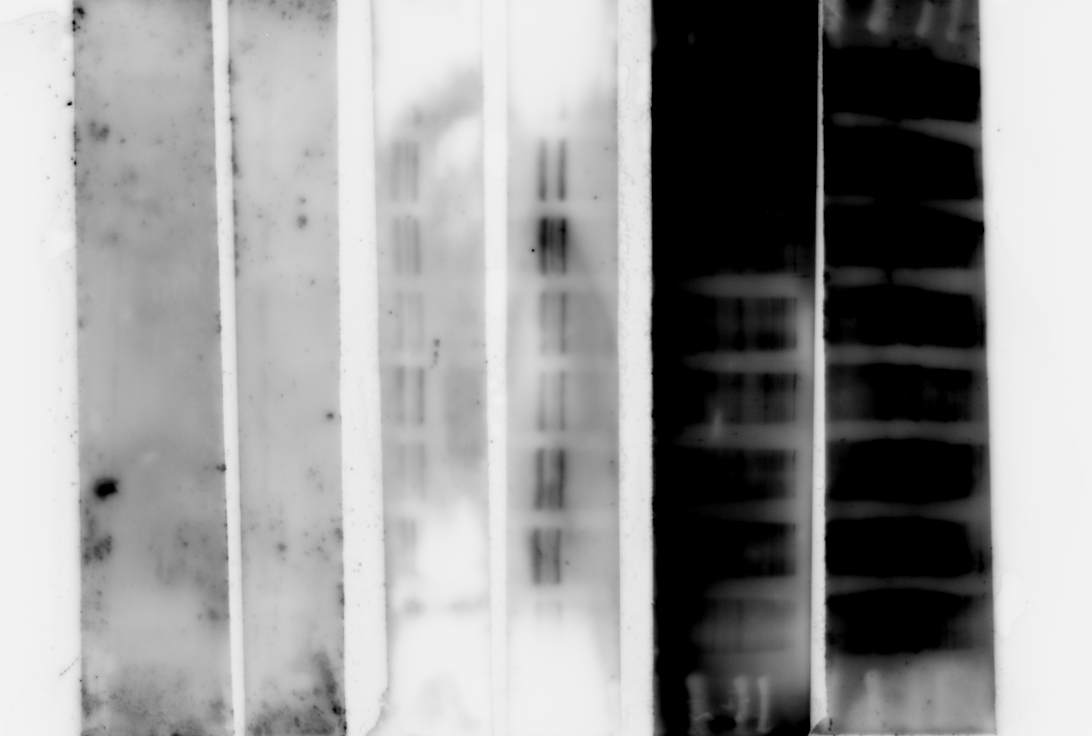
**

Vimentin

TGFβ1 (ng/ml)

20

10

0

**
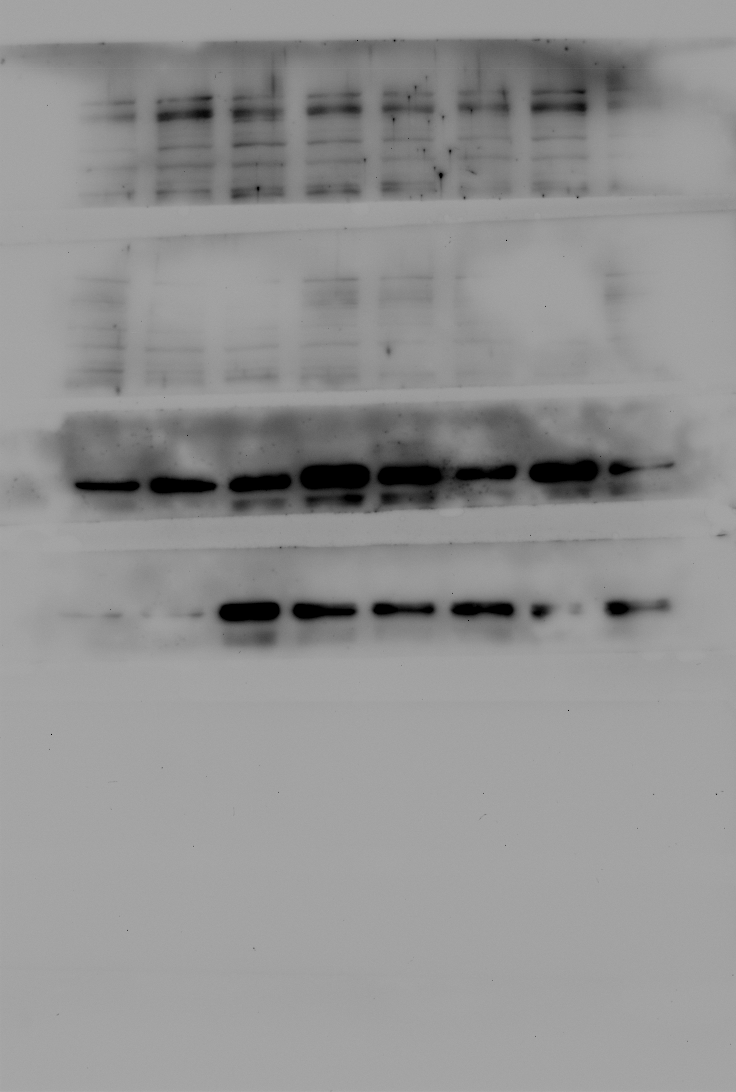
**

NCL

E-cadherin

**
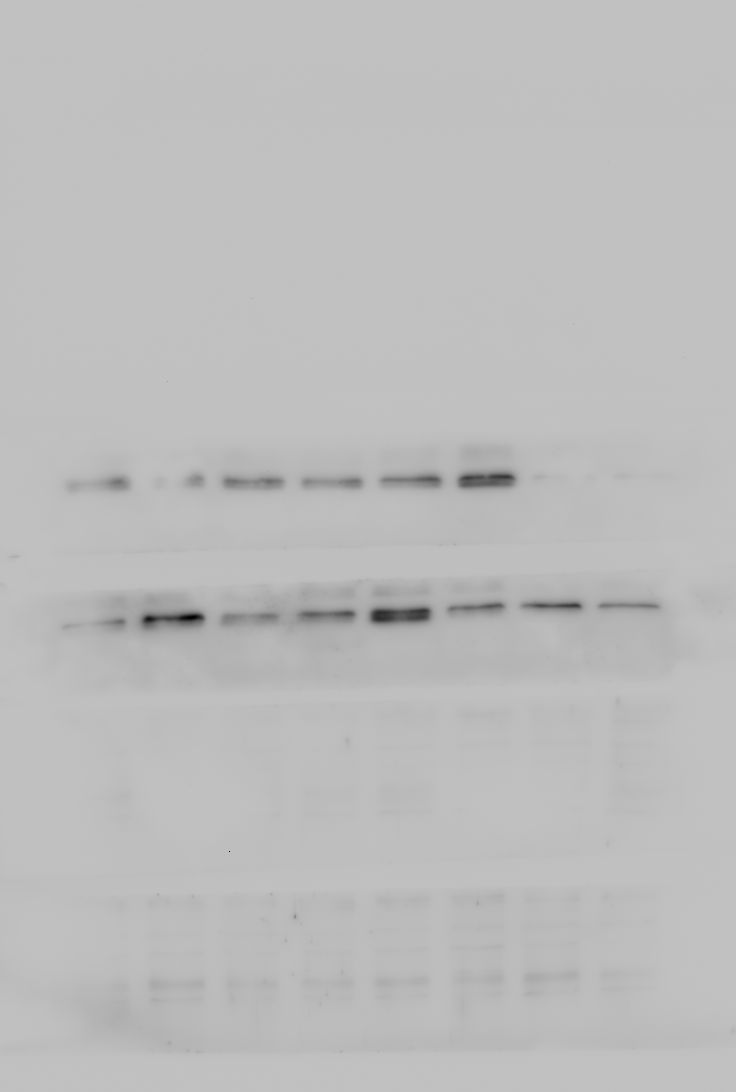
**

**
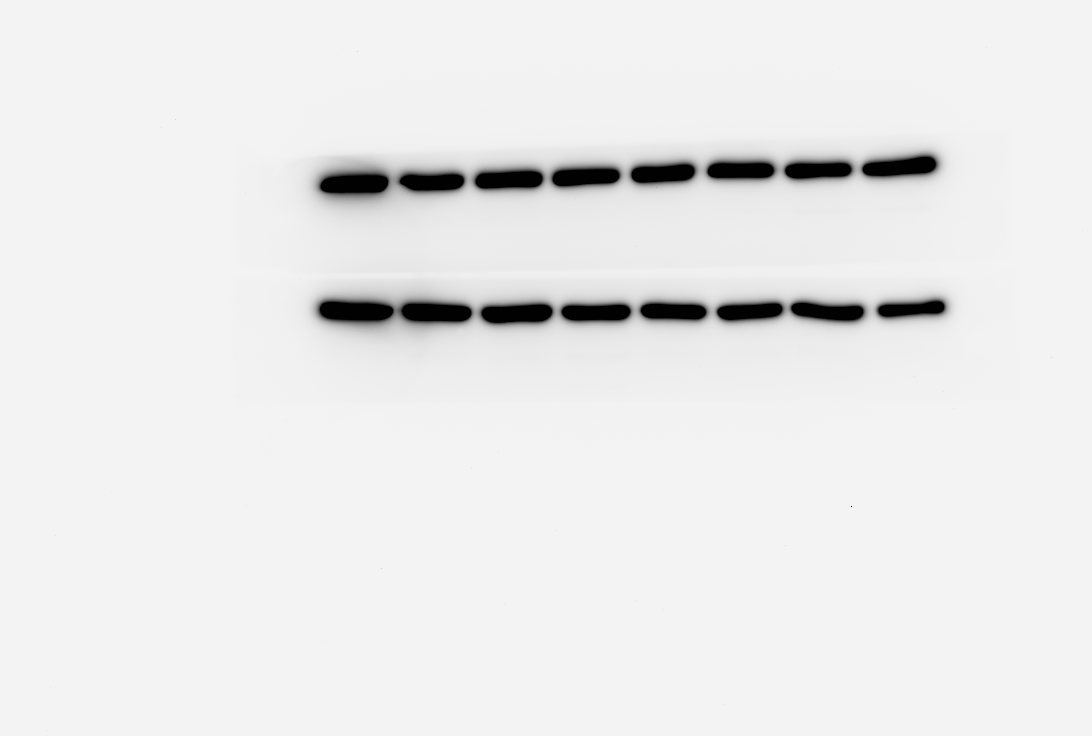
**

β-actin

**Figure S5**. The full-length blots of Figure S2 and all replicates. For immunoblotting, membranes were cut in terms of the molecular weight range prior to hybridization with primary antibody.

**Materials and methods**

**Cell growth curves**

SAS and SAS-EGFP-Fluc cells were seeded at a density of 3x10^4^ cells/dishes in 100 mm^2^ dishes.

After 24, 48, 72, 96 and 120 hr, the cells were detached using 0.25% trypsin-EDTA solution (Thermo Fisher Scientific Inc., Waltham, MA, USA). Cell counting was performed using 0.4% trypan blue solution and with a hemocytometer slide. Doubling time was analyzed and the growth curve was plotted using SigmaPlot12. Results were the mean ± standard deviation of triplicate experiments.

**Cell viability assay**

SAS-wt, SAS-EGFP-Fluc and SAS-E2-crimson-P2A::ttksr39 cells were seeded in a 96-well cell culture plate at the density of 5x10^3^ cells/well overnight and incubated with serial dilutions of Unc0642 (the concentration of Unc0642 was 0.625, 1.25, 2.5, 2, 5, 10, 20, 40, 80 μM respectively in culture medium). Plates were then incubated 48 hours at 37 °C under 5% CO_2_. Relative cell viabilities were determined by a AlamarBlue assay. 10% v/v of AlamarBlue (Serotec, Oxon, UK) was added into each well and the well containing culture medium without cells served as negative controls. Absorbance was measured at 570 nm against a reference wavelength at 600 nm using a microplate reader (Tecan, Infinite 200 pro, Zürich, Switzerland) after incubation for 24 h at 37°C. Results were presented as the percentage of AlamarBlue reduction and were corrected to background values of negative controls. IC_50_ values were calculated from a log([drug]) versus normalized response curve fit using SigmaPlot12.
